# Posterior reversible encephalopathy syndrome in Colombia a case series

**DOI:** 10.1038/s41598-026-38921-w

**Published:** 2026-02-07

**Authors:** Mónica Ortiz-Pereira, Mariana Gaviria-Carrillo, Isabella Esther Mendoza-Rodelo, Sofia Ramirez-Guerrero, María Alejandra Palacios-Ariza, Luis Roa-Wandurraga, Jesús Hernan Rodriguez Quintana, Camilo Romero

**Affiliations:** 1 Universidad del Rosario, Bogotá, Cundinamarca Colombia; 2 Hospital Universitario Mayor de Méderi, Bogotá, Cundinamarca Colombia; 3 Fundación Cardioinfantil, Bogotá, Cundinamarca Colombia; 4 Neuroscience Research Group (NeURos), Center for Neuroscience-NeURovitae, School of Medicine and Health Sciences, Universidad del Rosario, Bogotá, Colombia; 5 Undergraduate Neuroscience Research Group (SemineURos), Center for Neuroscience - NeURovitae, Universidad del Rosario, Bogotá, Colombia; 6 Unidad de Investigación, Fundación Universitaria Sanitas, Bogotá, Colombia

**Keywords:** Posterior reversible encephalopathy syndrome, PRES, Colombia, Case series, Diseases, Medical research, Neurology, Neuroscience, Risk factors

## Abstract

Posterior Reversible Encephalopathy Syndrome (PRES) is a rare clinical and radiological condition. There is limited information regarding the characteristics of PRES in the Colombian population. This study aims to describe the clinical-radiological characteristics of PRES in a series of cases in Colombia. A retrospective study was conducted between 2018 and 2024 with adult patients with PRES in two high complexity centers in Bogota, Colombia. Demographic, clinical, radiological, electroencephalographic, neurological complications and clinical outcomes were registered. Comparison of outcomes was performed between patients with and without neurological complications (ischemia, hemorrhage, or status epilepticus). A bivariate logistic regression analysis was performed to determine the risk factors associated with neurological complications of PRES. We included 60 patients with a median age of 55 years, 68.3% women. The most frequent risk/triggering factor was high systemic blood pressure (55%). Main symptoms reported were seizures (73.3%), impaired consciousness (60%), headache (43.3%) and visual changes (36.6%). Vasogenic cerebral edema was more common in the occipito-parietal region (33.3%). No fatalities were reported. The most frequent neurological complication was intracranial hemorrhage. Absence of complications was associated with shorter hospital stay (6 vs. 14 days). Additionally, a history of cerebrovascular disease and decreased visual acuity at presentation were associated with a lower observed rate of neurological complications following PRES (OR 0.1; 95% CI 0.01–0.91). Clinical-radiological characteristics of PRES in the Colombian population studied are similar to those described in literature. In our analysis, a history of cerebrovascular disease and decreased visual acuity at presentation were associated with a lower observed rate of neurological complications following PRES.

## Introduction

First described in 1996 by Hinchey et al.^1^, Posterior Reversible Encephalopathy Syndrome (PRES) is an uncommon clinical-radiological entity characterized by neurological symptoms and subcortical vasogenic cerebral edema^2,3^.

Mechanisms of disease in PRES are uncertain. Currently, two main theories have been proposed to explain the phenomena observed in this syndrome^4^. One involves endothelial dysfunction induced by circulating endogenous or exogenous toxins, and the other attributes the syndrome to endothelial injury caused by systemic blood pressure elevation^4,5^.

PRES affects both men and women across all age groups, though it is more frequent in women, with a mean age of 45 years at presentation^2–4^. A wide range of precipitating or risk factors for PRES have been reported, with elevated systemic blood pressure as the most prevalent. Other triggers include (pre)eclampsia, renal disease, hepatic dysfunction, chemotherapeutic agents, immunosuppressants, autoimmune disorders, and sepsis^2,3,6^.

Clinical manifestations of PRES typically have an acute onset and occur in temporal association with the precipitating factor. The most common symptoms include seizures (80%), headache (50%), visual disturbances (39%), altered mental status or encephalopathy (28%), and focal neurological deficits (10–15%)^7–9^.

Diagnosis of PRES is based on the presence of characteristic neurological symptoms, identification of a triggering factor, and the detection of subcortical vasogenic edema^2^. This edema commonly follows one of four imaging patterns: the occipito-parietal dominant pattern as the most frequent, holohemispheric watershed pattern, frontal predominant pattern, the central variant, or a combination of these^4,8,9^.

Management of PRES focuses on general medical care, withdrawal or control of the precipitating factors, and treatment of syndrome-related complications^4,10^. Although the overall prognosis is favorable, mortality can reach up to 6%^2,10^. The most frequent complications include status epilepticus, cerebral ischemia, hemorrhage, and intracranial hypertension^10–13^.

Despite increasing clinical recognition, PRES remains a relatively rare condition with limited global epidemiological data. Its heterogeneous presentation often leads to misdiagnosis and delayed treatment, which may increase the risk of complications. Most available studies originate from developed countries, rely on hospital registries, and are retrospective in nature.

In Latin America, particularly in Colombia, clinical, epidemiological, and radiological data on PRES is scarce. No multicenter case series have been published to date, and clinical manifestations, risk factors, and functional outcomes have not been systematically characterized in the local population. Limited context-specific clinical data contribute to delayed recognition and suboptimal management of PRES, particularly in tertiary centers caring for patients with high-risk comorbidities; accordingly, this study characterizes the clinical and radiological features of PRES in cases from two Colombian centers.

## Methods

### Patient identification

We retrospectively identified patients diagnosed with PRES between 2018 and 2024 from two institutional databases. The first database is a daily electronic hospitalization registry that captures demographic information, diagnoses, laboratory results, and imaging findings. The second database consists of the electronic medical record system and virtual imaging archives from both centers. Both databases have been fully operational and continuously updated since 2018.

We screened these sources for patients with diagnoses consistent with PRES or conditions within its clinical spectrum, including hypertensive encephalopathy, hypertensive emergency with neurological organ involvement, hypertension-related cerebral edema, drug-induced cerebral edema, cerebral endotheliopathy, eclampsia, HELLP syndrome, posterior reversible leukoencephalopathy, unspecified encephalopathy, cerebral hyperperfusion syndrome, metabolic or toxic encephalopathy, cerebral capillary leak syndrome, and other vascular encephalic syndromes.

Eligible patients were adults (> 18 years) who had been evaluated by a neurologist and exhibited clinical and radiological features typical of PRES, with neuroimaging interpreted by a neuroradiologist. We excluded patients with incomplete clinical or imaging data or those who had not been evaluated by a neurologist. PRES was diagnosed based on characteristic clinical symptoms (headache, visual disturbance, seizures, altered consciousness, or transient motor symptoms) together with standard radiological criteria—T2WI and FLAIR hyperintensities in subcortical or gyral regions—and confirmed by reversibility of clinical and imaging findings.

### Data collection

The data collection process was divided into the following phases:

1. **Review of the daily hospitalization registry** of the neurology department at each center to identify candidates with possible PRES.
2. **Review of the institutional medical records** of each candidate to verify the clinical inclusion and exclusion criteria.
3. **Review of neuroimaging studies** from each center’s diagnostic imaging archive to verify the imaging inclusion and exclusion criteria.
4. **Review of the institutional medical records** of each included subject to extract data for each study variable.
5. **Review of electroencephalogram reports** for each included subject.

Neuroimaging evaluation included T1WI, T2WI, and FLAIR sequences in all cases, with DWI/ADC, SWI or T2WI, contrast-enhanced T1WI, MRA, MRV, and other advanced modalities reviewed when available. DWI/ADC maps were used to distinguish vasogenic from cytotoxic edema, while SWI/T2WI assessed hemorrhage and microbleeds. MRI was the preferred modality, with CT performed only when MRI was not available. PRES was identified by characteristic vasogenic edema—manifesting as increased T2/FLAIR signal in the subcortical white matter, typically in parieto-occipital or posterior temporal regions, usually without T1 enhancement, or central-variant PRES, involving central structures such as the basal ganglia, thalamus, brainstem, and/or spinal cord^14^. Additional regions previously linked to PRES were also systematically evaluated. All imaging studies were independently reviewed by both a neurologist and a neuroradiologist.

To ensure consistency across records, key clinical variables were operationally defined. Hypertension was considered uncontrolled when Systolic Blood Pressure/Diastolic Blood Pressure was > 180/110–120 mmHg, or persistently above guideline thresholds despite treatment. Renal dysfunction was defined as an eGFR < 60 mL/min/1.73 m² or serum creatinine above the upper limit of normal. Eclampsia was diagnosed when seizures occurred in a pregnant or postpartum patient with preeclampsia and no alternative neurological explanation. Cytotoxic or immunosuppressive drug exposure was recorded when agents such as tacrolimus, cyclosporine, cyclophosphamide, or platinum-based therapies were administered within 30 days or at documented toxic levels.

Outcome measures included clinical complications (ischemia, hemorrhage, reversible vasoconstriction syndrome^14^, status epilepticus, or other), in-hospital mortality, 90-day readmission, hospital length of stay, discharge disposition, and functional status at discharge (independent in both basic and instrumental activities of daily living, independent in basic activities only, or dependent). For deaths, the cause was reviewed to determine whether it was PRES-related.

Sociodemographic and clinical data were recorded including past medical history, risk/triggering factors, body mass index (BMI), blood pressure at admission, labs at admission, clinical manifestations, neuroimaging findings, EEG findings, hospital re-entry, hospital stay duration, and neurologic complications related to PRES.

### Ethical considerations and reporting standards

This study was conducted in accordance with Colombian national guidelines for health research (Resolution 8430 of 1993 and Law 23 of 1981). The ethics and research committees of both La Cardio and Hospital Mayor Mederi approved the study. Given the retrospective design, minimal risk, and absence of patient intervention, both committees waived the requirement for informed consent.

Each patient was registered in REDCap using a unique code to ensure anonymity. No identifying personal data were stored in any study instrument. Extracted data was stored on secure, access-restricted computers belonging to the principal investigator and co-investigators. Document review, data extraction, instrument creation, and transcription were performed exclusively by researchers authorized under the study protocol. The principal investigator was responsible for safeguarding all information, ensuring participant anonymity and data privacy, maintaining restricted access, and guaranteeing the security of both manual and electronic records generated throughout the investigation. All documents were securely archived and accessible only to study personnel and authorized individuals.

### Data analysis

According to their distribution, continuous variables were reported either as means and standard deviations or as medians and interquartile ranges. Categorical variables were reported as frequencies and percentages. Patients were divided in two groups: patients with neurologic complications due to PRES and patients without any neurologic complication. The Chi-squared and Fisher’s exact test were performed for qualitative variables, whilst the T-student and U Mann-Whitney tests were conducted for continuous variables depending on their distribution. Bivariate analyses were performed to determine the risk factors associated with neurologic complications of PRES. Odds ratios (OR) were calculated with a 95% confidence interval (CI) for each risk factor as identified in the logistic regression model. Statistical significance was considered at *p* < 0.05. Statistical analysis was performed using StataCorp. Version 15.0. Stata: Statistical Software.

## Results

During the study period, 60 adult patients with PRES were included with a median age of 55 years (IQR 32–69). There were 41 (68.3%) females, five (8.3%) of which were pregnant and 5 (8.3%) had a history of preeclampsia. Past medical history included systemic hypertension (55%), chronic kidney disease (48.3%) and autoimmune disease (36.7%) (Table 1). Three (5%) of the patients with autoimmune disease were diagnosed with Systemic Lupus Erythematosus (SLE). Three patients had active SARS-CoV2 infection, however only one considered it a triggering factor for PRES. Table 2 summarizes the etiologies associated with PRES. Subjects could present with one or more etiological categories; therefore, the individual count for each category was reported. Mean arterial blood pressure at admission was 177 mmHg, with 22 (36.6%) patients with values above 180 mmHg and 29 (48.3%) diagnosed with hypertensive emergency.

**Table 1:** Demographic characteristics, hospital stay duration, and past medical history of patients with PRES in two high complexity centers: Bogotá, Colombia (2018–2024).

Variable	Total (*n* = 60)	With neurologic complication (*n* = 31)	Without neurologic complication (*n* = 29)	*p*-value
Age (years) Median [IQR]	55 [32, 69]	56 [33.5, 68.5]	48 [26, 69]	0.610
Hospital stay (days) Median [IQR]	7 [ 5, 20]	14 [ 7, 27]	6 [4.0, 7.5]	**0.008**
	n (%)			
Sex				0.585
Female	41 (68.3)	20 (64.5)	21 (72.4)	
Male	19 (31.7)	11 (35.5)	8 (27.6)	
Reason for admission				0.874
Primary for PRES	42 (70.0)	23 (74.2)	19 (65.5)	
Remission for PRES	2 (3.3)	1 (3.2)	1 (3.4)	
Primary for other cause	11 (18.3)	5 (16.1)	6 (20.7)	
Remission for other cause	5 (8.3)	2 (6.5)	3 (10.3)	
Past medical history				
Hypertension	33 (55.0)	16 (51.6)	17 (58.6)	0.614
CKD	29 (48.3)	16 (51.6)	13 (44.8)	0.617
Autoimmune disease	22 (36.7)	11 (35.5)	11 (37.9)	1
Other cardiopathies	8 (13.3)	5 (16.1)	3 (10.3)	0.708
Diabetes	7 (11.7)	5 (16.1)	2 (6.9)	0.426
Coronary disease	7 (11.7)	5 (16.1)	2 (6.9)	0.426
Neoplasia	5 (8.3)	3 (9.7)	2 (6.9)	1
TIA/Stroke	4 (6.7)	0 (0.0)	4 (13.8)	**0.049**
Solid organ transplant	2 (3.3)	1 (3.2)	1 (3.4)	1
None	10 (16.7)	5 (16.1)	5 (17.2)	1
Past surgical history				0.563
None	31 (51.7)	14 (45.2)	17 (58.6)	
Heart surgery	1 (1.7)	1 (3.2)	0 (0.0)	
Lung surgery	14 (23.3)	9 (29.0)	5 (17.2)	
Lung transplant	14 (23.3)	7 (22.6)	7 (24.1)	
Previous modified Rankin score				0.509
0	48 (80.0)	23 (74.2)	25 (86.2)	
1	9 (15.0)	6 (19.4)	3 (10.3)	
2	3 (5.0)	2 (6.5)	1 (3.4)	

Significant values are in [bold].

p-values: continuous variables correspond to U Mann-Whitney test, qualitative variables correspond to Fisher ‘s exact test, IQR: Interquartile Range. CKD: Chronic Kidney Disease, TIA: Transient Ischemic Attack.

**Table 2:** Etiologies associated with PRES.

Etiology	Counts	% of Total
Unknown	1	1.1%
Autoimmune disease	11	12.6%
Upper GI bleeding	1	1.1%
Not Reported	1	1.1%
Raised blood pressure	48	55.2%
Acute infection	4	4.6%
Hepatic disease	3	3.4%
Eclampsia	3	3.4%
COVID-19	4	4.6%
Hyperglycemia	1	1.1%
Heart failure	1	1.1%
Renal disease	1	1.1%
Immunosuppression	2	2.3%
Pregnancy	1	1.1%
Symptomatic anemia	1	1.1%
Post-surgical	1	1.1%
Lung transplant	1	1.1%
Neoplasia	1	1.1%
Drug toxicity	1	1.1%

Symptom duration prior to admission ranged from 1 to 11 days. The most frequent clinical manifestation was epileptic seizures in 44 (73.3%) patients, most of these were focal to bilateral tonic-clonic seizures or with a generalized onset. 36 (60%) patients reported impaired consciousness, 8 (13.4%) with stupor or in coma. 26 (43.3%) patients reported headache, with the tension-type headache as the most frequent phenotype. Visual symptoms were indicated by 22 (36.6%) patients, with blurry vision as the most frequent (Table 3).

**Table 3:** Clinical characteristics of patients with PRES in two high complexity centers: Bogotá, Colombia (2018–2024).

Variable	Total (*n* = 60)	With neurologic complications (*n* = 31)	Without neurologic complications (*n* = 29)	*p*-value
	Median (IQR)			
Systolic blood pressure (mmHg)	177 [161, 200]	177 [151, 200]	180 [170, 200]	0.654
BMI (kg/m2)	23.2 [19.5, 26.1]	23.7 [19.4, 27.7]	23.0 [19.9, 24.1]	0.609
Creatinine (mg/dl)	1.5 [0.9, 7.0]	1.3 [0.8, 8.1]	2.7 [0.9, 6.7]	0.512
BUN (mg/dl)	30 [18, 50]	30 [19, 44]	31 [17, 51]	0.806
Hemoglobin (mg/dL)	12.4 [9.4, 14.1]	12.3 [10.4, 14.1]	12.6 [9.3, 14.0]	0.857
Platelets (10^3 cells/mm3)	216 [158, 304]	248 [174, 335]	200 [150, 258]	0.091
	n (%)			
Hypertensive emergency				
Yes	29 (48)	11 (41)	18 (53)	0.312
No	31 (52)	16 (59)	15 (44)	
Level of consciousness				0.788
Alert	24 (40.0)	11 (35.5)	13 (44.8)	
Confusion	12 (20.0)	5 (16.1)	7 (24.1)	
Somnolence	15 (25.0)	9 (29.0)	6 (20.7)	
Stupor	7 (11.7)	4 (12.9)	3 (10.3)	
Coma	1 (1.7)	1 (3.2)	0 (0.0)	
Other	1 (1.7)	1 (3.2)	0 (0.0)	
Headache				
Tension-type	20 (33.3)	6 (19.4)	14 (48.3)	**0.028**
Migraine	8 (13.3)	3 (9.7)	5 (17.2)	0.465
Thunderclap	5 (8.3)	4 (12.9)	1 (3.4)	0.355
Epileptic seizures				0.975
No seizures	16 (26.7)	9 (29.0)	7 (24.1)	
Focal	6 (10.0)	3 (9.7)	3 (10.3)	
Generalized	32 (53.3)	16 (51.6)	16 (55.2)	
Epileptic status	6 (10.0)	3 (9.7)	3 (10.3)	
Visual symptoms				
None	38 (63.3)	23 (74.2)	15 (51.7)	0.108
Blurry vision	15 (25.0)	5 (16.1)	10 (34.5)	0.139
Decreased visual acuity	8 (13.3)	1 (3.2)	7 (24.1)	**0.024**
Hemi/Quadrantanopia	5 (8.3)	1 (3.2)	4 (13.8)	0.188
Other	2 (3.3)	1 (3.2)	1 (3.4)	1.000
Visual negligence	1 (1.7)	0 (0.0)	1 (3.4)	0.483
Hospital re-entry after PRES				
No	50 (83.3)	27 (87.1)	23 (79.3)	0.500
Yes	10 (16.7)	4 (12.9)	6 (20.7)	
Electroencephalographic study				
None	24 (40)	12 (39)	12 (41)	0.765
EEG	3 (5)	1 (3)	2 (7)	
VEEG	33 (55)	18 (58)	15 (52)	

Significant values are in [bold].

p-value for continuous variables corresponds to the U Mann-Whitney test and the Fisher exact test for qualitative variables. BMI: Body Mass Index, BUN: Ureic nitrogen in blood, EEG: Electroencephalogram, VEEG: Video Telemetry.

**Fig. 1:**
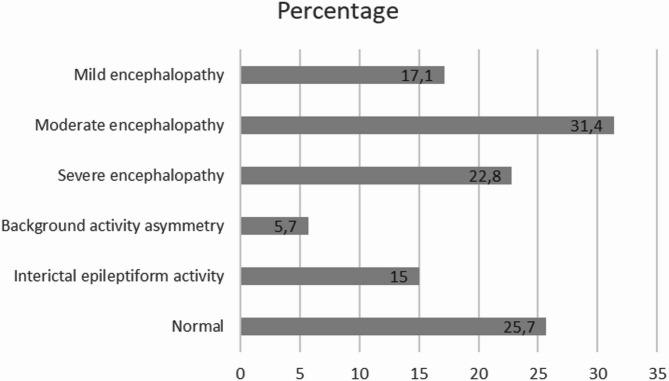
EEG patterns.

Thirty-five EEG studies were conducted in patients with PRES. Of these, 9 (25.7%) were normal, 25 (71.4%) showed signs of encephalopathy, 9 (15%) evidenced interictal epileptiform activity and 2 (5.7%) showed background activity asymmetry. Encephalopathy was considered severe in 8 (22.8%) cases, moderate in 11 (31.4%), and mild in 6 (17.1%) (Fig. 1). Interictal epileptiform activity was shown as lateralized periodic discharges (LPD) in 4 cases, as spike-waves in 4, focal sharp waves in 4 and one case with bilateral independent LPDs.

MRI was used for diagnosis in 95% of cases. One third of patients reported occipito-parietal cerebral edema, 7 (11.7%) occipital only, and 6 (10%) cases with both an occipital and temporal-insular localization. Infratentorial edema was found in 10 (16.6%) patients, eight of which were reported in addition to supratentorial edema. Vasogenic cerebral edema at the basal ganglia was found in only one patient.

The most frequent neurologic complication was intracranial hemorrhage, specifically 16.7% intracerebral hemorrhage and 16.7% subarachnoid hemorrhage. Asymptomatic infarction was found in 15% of patients, and convulsive epileptic status in 11.7% (Table 4). Median hospital stay duration was longer in patients with neurologic complications (14 days, IQR 7–27) compared to those without neurologic complications (6 days, IQR 4-7.5). Past medical history of Transient Ischemic Attack (TIA) or Stroke was evidenced in four patients without neurologic complications (*p* = 0.049). Decreased visual acuity was the most frequent manifestation in patients without neurologic complications (24.1% vs. 3.2%), with a statistically significant association (OR 0.1 IC 95% 0.01–0.91; *p* = 0.024) (Tables 1 and 3).

**Table 4:** Neurological complications of patients with PRES in two high complexity centers: Bogotá, Colombia (2018–2024).

Neurological complications	*n* (%)
Asymptomatic infarction	9 (15.0)
Symptomatic ischemia	1 (1.7)
Intracerebral hemorrhage	10 (16.7)
Subarachnoid hemorrhage	10 (16.7)
RPVS	2 (3.3)
Epileptic status	7 (11.7)
Other	1 (1.7)
None	29 (48.3)

All patients were functionally independent prior to PRES diagnosis, 48 (80%) had a modified Rankin scale (mRS) score of zero, and 12 (20%) patients had scores of 1 and 2. Thirteen (21.6%) patients showed functional deterioration at hospital discharge. Functional deterioration was more frequent in males (*n* = 8, 42.11%) compared to females (*n* = 5, 12.2%), with a significant association (OR 5.24 IC 95% 1.42–19.32; *p* = 0.0164).

The median duration of hospital stay was 30 days (IQR 18.5–30.8) in patients with functional deterioration compared to 7 days (IQR 5.0-8.8) in patients without functional deterioration with a statistically significant difference (*p* = 0.0009). Two patients reported functionality improvement when compared to functional status prior to the PRES diagnosis. No deaths were reported among the patients with PRES included in this study.

## Discussion

### Epidemiology, risk factors, and etiology

Knowledge about the incidence and clinical impact of PRES, a rare neurological condition, has progressively increased. One of the most recent and robust descriptions of its epidemiology is a study conducted in the United States, reporting a national incidence of approximately 3 cases per 100,000 people per year between 2016 and 2019^9^. Other reports in the literature cite an incidence of 2.7–25% in bone marrow transplant patients, 0.4% in solid organ transplant recipients, 0.84% in end-stage renal disease, and 0.69% in patients with SLE, reflecting higher prevalence among critically ill or immunocompromised populations^3^. In Latin America, demographic data is lacking; most information comes from small case series or case reports^12^.

Our findings evidence a more frequent compromise of PRES in females around 55 years old, which is consistent with previous literature^15^. However, in regional data, the available evidence remains limited, as much of it is derived from case reports or small case series and includes the pediatric population^12,16,17^.

The most commonly reported risk factor for PRES is a rapid elevation in blood pressure^18^, followed by renal disease, autoimmune disorders, and preeclampsia/eclampsia^15^. Hypertension is both a common risk and the most frequent precipitating factor for PRES. In our series, 55% of patients had a history of hypertension, and 48.3% presented with a hypertensive crisis. Hypertension prevalence varies across populations; for instance, in the U.S., African American individuals are more affected, due to socioeconomic, genetic, and cultural factors^19,20^. In Colombia, similar disparities have been linked to socioeconomic and educational variables, though our sample size and available data limited further analysis.

In our study, besides hypertension, chronic kidney disease (48.3%) and autoimmune disorders (36.7%) were notably prevalent as the second and third most prevalent risk factors, respectively. Organ transplantation was also identified as a risk factor—likely reflective of the patient complexity at our centers. We also identified three cases of PRES related to SARS-CoV-2 infection—one without comorbidities and two presenting with hypertensive crisis. During the pandemic, PRES has been reported as a CNS complication of COVID-19^5,20,21^, likely triggered by cytokine storms and endothelial dysfunction. These cases suggest the interplay of both hyperperfusion and inflammatory mechanisms, possibly in a synergistic “threshold effect.”

Additionally, many patients had a history of tobacco, alcohol, or psychoactive substance use. We propose the hypothesis that chronic endothelial injury from exogenous substances may contribute to PRES risk in the general population. Autoimmune diseases emerged as the second most common risk factor in our series, suggesting a possible inflammatory role in PRES pathogenesis, though we lacked data on immunosuppressive treatment or disease activity at presentation. 

Our findings reaffirm arterial hypertension as the most prevalent risk factor and hypertensive crisis as a common precipitating event. Moreover, systemic autoimmune diseases may be underrecognized contributors, warranting active investigation in PRES cases. Notably, in bivariate analysis of our cohort, a history of stroke or TIA and decreased visual acuity were associated with a lower probability of neurological complications. Decreased visual acuity was statistically significant, possibly indicating earlier diagnosis or reduced association with RCVS. A history of stroke/TIA may reflect altered autoregulation or protective vascular adaptations. Other frequently reported etiologies include the use of immunosuppressive drugs such as oral corticosteroids, mycophenolate, cyclosporine, and cyclophosphamide. In this regard, Pirola et al.^17^ reported that PRES was associated with the use of oral steroids in 4 patients (31%), tacrolimus in 4 (31%), mycophenolate mofetil in 3 (23%), azathioprine in 3 (23%), cyclosporine in 3 (23%), hydroxychloroquine in 2 (15%), cyclophosphamide in 2 (15%), and rituximab in 1 patient (8%).

Another etiology that should be explored is the use of psychoactive substances, such as LSD and cocaine, as well as toxic exposures, including scorpion envenomation. Additionally, sepsis—through its association with endothelial dysfunction—represents a frequent contributing factor in patients with PRES^15^.

### Pathophysiology

The exact pathophysiology of PRES remains unclear; however, it is a syndrome with a dysregulation of brain perfusion, causing vasogenic edema. Two primary theories have been proposed: one involving physical stress from elevated blood pressure and hyperperfusion, and another centered on inflammation-mediated endothelial dysfunction^7,11^.

One of the proposed pathophysiological theories of PRES suggests that rapid increases or significant fluctuations in blood pressure exceeding the upper limit of cerebral autoregulation may result in cerebral hyperperfusion^22^. When blood pressure rises abruptly and severely, the autoregulatory response becomes insufficient, leading to hyperperfusion, endothelial dysfunction, disruption of the blood–brain barrier (BBB), and extravasation of plasma and macromolecules into the cerebral parenchyma^22^. Supporting the hyperperfusion model, perfusion MRI studies have shown significantly higher cerebral blood flow in affected versus unaffected brain regions (median 100.4 vs. 61.0 ml/100 g-min; *p* < 0.001)^23^. Posterior brain regions are particularly susceptible to hyperperfusion due to their relatively lower sympathetic innervation^22^.

Additional clinical scenarios associated with acute blood pressure elevation or marked fluctuations include dysautonomia, such as those observed in patients with Guillain–Barré syndrome. These observations support the vasogenic edema theory, which postulates that rapid increases or variability in blood pressure overwhelm cerebral autoregulation, resulting in BBB disruption and subsequent vasogenic edema^24^.

Not all patients with PRES have acute changes in blood pressure and immune dysregulation and systemic diseases have been shown to trigger PRES. These disorders may cause vascular leakage and edema through the release of cytokines including tumor necrosis factor alpha (TNF alpha), interleukin-1 (IL-1) and interferon gamma. These cytokines activate the endothelial release of vasoactive factors, promote leucocyte-endothelial cell interaction, and can influence the expression of genes that increase vascular permeability and cause interstitial edema. Elevated levels of vascular endothelial growth factor (VEGF) have been associated with the development of PRES. VEGF increases vascular permeability, TNF alpha induces its expression on endothelial cells, with higher levels observed in patients with SLE and PRES compared to healthy controls.

Autoimmune disease, the third most common risk factor in this study, has also been implicated in PRES^25^. In SLE patients, PRES prevalence ranges from 0.43% to 2.0%^25^. Proposed mechanisms include elevated cytokines—TNF-α, IL-1, IL-6—which are neurotoxic and promote endothelial dysfunction via VEGF and endothelin-1 (ET-1).

### Clinical manifestations

The most common clinical findings in our cohort were seizures (73.3%), encephalopathy (60%), headache (43.3%), and visual disturbances (36.6%)—consistent with prior literature^2,11^. Tonic-clonic seizures were the most frequent. 10% of patients were diagnosed with status epilepticus. Somnolence was noted in 25%, tension-type headache was the most common, and blurred vision was reported in 25%. These findings align with other reports^16,26^, although milder or atypical manifestations may have been underreported due to diagnostic bias.

Previous studies report status epilepticus in up to 13% of cases^27^. In an Indian cohort of 40 patients, 77.5% had seizures and 8 developed status epilepticus; EEG showed generalized slow spike-wave discharges in all patients^23^. Our EEG findings were more diverse: diffuse/focal slowing, epileptiform activity, asymmetries, lateralized periodic discharges, and some normal traces.

The pathophysiological mechanism of seizures in PRES likely involves cortical irritation due to vasogenic edema and BBB disruption. No specific EEG pattern has been established; some mimic autoimmune or metabolic conditions^13^, while posterior epileptiform discharges may be suggestive of PRES^8^.

High blood pressure is a feature of most PRES patients, regardless of the trigger factor. Hypertension may aggravate the clinical condition and lowering the blood pressure is associated with clinical and radiological recovery. Specific blood pressure management protocols for PRES patients have not been developed. The medications used and treatment goals should be based on known hypertension protocols, prior blood pressure levels, individual characteristics, and clinic-radiological recovery. Close follow-up of blood pressure is warranted. We recommend resetting the goals according to the clinical condition of the patient and maybe to the results obtained through non-invasive methods of blood flow measurement such as transcranial doppler or perfusion imaging^23^.

There are many clinical acute neurological conditions that have similar manifestations (headache, vision changes, seizures, and delirium) to PRES. Stroke, encephalitis, toxic/metabolic encephalopathies, demyelinating diseases, cerebral venous thrombosis, and complicated migraine are amongst the most common. Key differential features are the clinical context, the MRI findings and laboratory work up. The presence of a PRES trigger related factor, posterior subcortical bilateral vasogenic edema and relatively normal work-up favor the diagnosis of the syndrome. Remembering that stroke can be a PRES complication is crucial to the proper diagnostic work-up and treatment strategies. On the other hand, some diagnoses may have an intimate relation to or are part of the spectrum of PRES: Hypertensive encephalopathy, eclampsia, and RCVS^28^.

### Neuroimaging

MRI is crucial for diagnosing PRES. Typical neuroimaging findings in PRES include bilateral T2/FLAIR hyperintensities without diffusion restriction, predominantly affecting the parieto-occipital or posterior frontal regions. These findings, observed in more than 70% of cases, are consistent with vasogenic edema^29^. Vasogenic edema has been described in both corticosubcortical regions and in a central variant. The most common corticosubcortical patterns include involvement of the parieto-occipital lobes (22%), a holohemispheric watershed pattern (23%), and the superior frontal sulcus pattern (27%). Mixed patterns involving the frontal and temporal lobes are also frequently reported^15,17^. The central variant is characterized by involvement of the brainstem, basal ganglia, posterior limb of the internal capsule, cerebellum, and periventricular regions, without cortical or subcortical involvement. Post-contrast enhancement has been described in 38–50% of cases and may present as a leptomeningeal pattern, a cortical pattern within areas of edema, or a combined pattern^15^. Intracranial hemorrhage, including microhemorrhages, intraparenchymal hemorrhage, and subarachnoid hemorrhage, has been reported in 10–25% of patients with PRES, which is in line with our results.

### Prognosis

Prognosis is usually benign in most PRES case series. Full recovery is expected within a week, MRI resolution occurs in more than two thirds of patients and mortality is around 3% to 6%. Sequelae reported in 10 to 20% of cases are related to ischemic or hemorrhagic complications, neuroimaging recovery lags clinical improvement and death may result from severe edema, intracranial bleeding, or the underlying clinical condition^14^. While 78.3% of our patients had favorable outcomes (mRS 0–2), 21.7% had unfavorable outcomes (mRS 3–5), which were statistically more frequent in men (OR 5.24; 95% CI 1.42–19.32). Neurological complications were associated with longer hospital stays (14 vs. 6 days). Intracranial hemorrhage (33.4%) was the most common complication, followed by cerebral infarction (15%) and status epilepticus (11.7%). Despite this, no in-hospital deaths occurred. Outcomes in PRES are probably related to the underlying condition, time to treatment, and imaging findings. Despite the persistence of related underlying conditions or trigger factors, PRES recurrence is not frequent, and epilepsy appears to be a rare clinical consequence of the syndrome^14^.

In our analysis, a history of cerebrovascular disease and decreased visual acuity at presentation were associated with a lower observed rate of neurological complications following PRES. Patients with TIA/stroke are typically under closer surveillance and monitoring, which may lead to earlier and more aggressive interventions, including prompt management of vascular risk factors. In addition, decreased visual acuity, a characteristic feature of PRES, may facilitate earlier recognition of the syndrome. Although this association was observed, this is a case series study and therefore does not allow causal inferences. Accordingly, these findings should be interpreted with caution.

Hypertensive urgency has been linked to lower mortality^14^. Hemorrhage risk is higher in patients with coagulopathy or thrombocytopenia^30–32^, though such data were unavailable in our study. The absence of fatalities in our cohort may reflect early diagnosis and appropriate management, but further prospective studies are needed. However, alternative explanations such as selection bias, underdiagnosis, or misdiagnosis should also be considered. Given that information was collected from medical records, there was a high risk of bias based on incomplete, missing, or inaccurate data. In addition, cases were selected based on data availability and typical features of the condition and cases were only recruited from two centers, all of which may have introduced a selection bias.

To our knowledge, this is the largest reported case series of PRES in Colombia, comprising 60 patients over six years at two high-complexity centers. The clinical, laboratory, and imaging profiles were largely consistent with international data, particularly the cohorts of Fugate et al.^14^ and Otite et al.^9^. However, distinctive features in our cohort included a high frequency of intracranial hemorrhage, notable autoimmune and substance-related risk factors, and an absence of hospital mortality. These findings underscore the value of studying PRES in diverse clinical and epidemiologic contexts. Early diagnosis, appropriate imaging, and expanded multicenter studies are key to improving our understanding and management of PRES in Latin America.

### Strengths and limitations

PRES, although an uncommon clinic-radiological condition, has been described in both sexes, different countries, varying ethnicities, and diverse populations. However, most large case series include mainly non-Latino populations.

The study may open avenues for future investigations of this condition in our yet understudied population. To the best of our knowledge this study is the largest case series of PRES patients in Colombia and one of the largest in Latin America. As shown in other case series, women were more commonly affected with headaches, seizures, altered mental status and visual complaints as the main clinical manifestations.

## Data Availability

Data will become available upon reasonable request. Contact: mariana.gaviria@urosario.edu.co.
